# A Short-Term Traffic Flow Prediction Method Based on Personalized Lightweight Federated Learning

**DOI:** 10.3390/s25030967

**Published:** 2025-02-06

**Authors:** Guowen Dai, Jinjun Tang

**Affiliations:** Smart Transport Key Laboratory of Hunan Province, School of Transport and Transportation Engineering, Central South University, Changsha 410075, China; 224201010@csu.edu.cn

**Keywords:** traffic flow prediction, urban land planning, federated learning, personalization, model pruning

## Abstract

Traffic flow prediction can guide the rational layout of land use. Accurate traffic flow prediction can provide an important basis for urban expansion planning. This paper introduces a personalized lightweight federated learning framework (PLFL) for traffic flow prediction. This framework has been improved and enhanced to better accommodate traffic flow data. It is capable of collaboratively training a unified global traffic flow prediction model without compromising the privacy of individual datasets. Specifically, a spatiotemporal fusion graph convolutional network (MGTGCN) is established as the initial model for federated learning. Subsequently, a shared parameter mechanism of federated learning is employed for model training. Customized weights are allocated to each client model based on their data features to enhance personalization during this process. In order to improve the communication efficiency of federated learning, dynamic model pruning (DMP) is introduced on the client side to reduce the number of parameters that need to be communicated. Finally, the PLFL framework proposed in this paper is experimentally validated using LPR data from Changsha city. The results demonstrate that the framework can still achieve favorable prediction outcomes even when certain clients lack data. Moreover, the communication efficiency of federated learning under this framework has been enhanced while preserving the distinct characteristics of each client, without significant interference from other clients.

## 1. Introduction

Traffic flow prediction has always been a hot topic in the field of transportation research. Traffic flow prediction plays a crucial role not only in optimizing transportation systems but also as a powerful tool for monitoring urban expansion, land use changes, and infrastructure development. By analyzing traffic flow patterns, urban planners can identify areas of city expansion and resulting land use changes. This insight aids in adjusting land use policies and optimizing urban development plans to better support sustainable urban growth. Traffic dynamics reflect the spatial and temporal distribution of human activities, serving as a key indicator for land monitoring. Traffic flow data can help create heat maps of human activity, providing real-time data support for urban planning. This connection not only aids in understanding current land use efficiency but also in predicting future land demand and change trends, offering a basis for policy making. After in-depth research by numerous scholars and decades of development, prediction methods have evolved from simple mathematical statistics in the early days to the various machine learning models currently available. However, short-term traffic flow prediction still faces many challenges. With the rise of machine learning and big data technology, the role of historical traffic flow data in short-term traffic flow prediction is becoming increasingly prominent. However, there are still some issues in the field of traffic flow prediction at present [1,2,3].

1. A significant factor limiting the further improvement of prediction accuracy is the low quality of traffic data. Most cities rely on data collected from intersection cameras, while only a few have deployed specialized equipment such as magnetic or sensor loops on specific roads to gather traffic flow data. Consequently, traffic data in many areas suffer from varying degrees of missing information or other quality issues. Under these circumstances, the performance of traditional centralized training models is greatly constrained.
2. Data privacy concerns are also a critical issue; traffic data from different cities or data collection agencies are often not transparent to each other. This leads to situations where a single dataset from one agency is insufficient to train a prediction model to an adequate level of excellence. If it were possible to collaboratively train traffic flow prediction models without revealing each other’s data, this could significantly increase the amount of training data, thereby enhancing the model’s predictive capabilities. This is particularly significant for cities or regions with limited data. However, the current high confidentiality requirements of cities and data agencies are limiting data sharing and training, thus necessitating a solution to the related data privacy and confidentiality issues.
3. With the continuous development of machine learning models, the models applied to short-term traffic flow prediction have become increasingly complex, and scholars tend to combine and stack machine learning models. While this approach can improve prediction accuracy to some extent, it also increases model complexity and computational burden, which in turn reduces computational efficiency.

In response to the challenges and difficulties currently faced in the field of traffic flow prediction, we propose a personalized lightweight federated learning framework (PLFL) for short-term traffic flow prediction. Our contributions can be outlined as follows.

1. We introduce a dynamic model pruning strategy that performs pruning on the local models of the clients. This approach reduces the number of model parameters that need to be uploaded, thereby enhancing the communication efficiency of federated learning.
2. We propose a personalized federated learning method. In practice, clients vary in the amount of data they possess, which leads to discrepancies in their influence on the model parameters during local training. Clients with substantial data can disproportionately impact the final global model, compromising the personalization of other clients and creating an unfair situation. To rectify this, we incorporate a feature attention mechanism into the global model parameter aggregation phase. When the server aggregates the model parameters uploaded by the clients, it calculates the distance between the current local model parameters, and the pre-processed global model parameters are calculated to ascertain the differences between models. Based on these differences, we customize different weights for each client’s model to enhance their individuality. Afterward, these weights are averaged to determine the final global model parameters, ensuring that the unique characteristics of each model are preserved while they learn from one another.
3. We propose a spatiotemporal fusion model that integrates a graph attention network (GAT), a graph convolutional network (GCN), and a multi-head temporal convolutional network (MH-TCN). Initially, MH-TCN is utilized to extract temporal features from the time series data, capturing both local and long-range temporal dependencies. Subsequently, a multi-head GAT is employed to compute attention scores for the relationships between road nodes, weighting the spatial neighbor subgraphs at different time points. The GCN is then used to extract spatial correlations from the dynamically weighted subgraphs.

The remainder of this paper is structured as follows: Section 2 provides an overview of related work in traffic flow prediction research and federated learning. Section 3 presents the methodology, offering a detailed explanation of the approach proposed in this paper. Section 4 describes the data used in this study. Section 5 is dedicated to the experimental analysis, while Section 6 concludes this paper with a summary of the findings and a discussion on future perspectives.

## 2. Related Work

In recent years, various machine learning models have been successively applied to short-term traffic flow prediction, including classical models such as long short-term memory (LSTM) networks and convolutional neural networks (CNNs). Federated learning, on the other hand, is an emerging machine learning paradigm that enables model training without the need to share data. First proposed by Google in 2016, it has experienced rapid development over the years and has become one of the hot research directions in the field of machine learning.

### 2.1. Federated Learning

With the development of intelligent transportation systems, the scale and complexity of traffic data are continuously increasing, and privacy and security concerns are also gaining more attention [4]. In this context, federated learning, as a distributed machine learning paradigm, can effectively address data privacy and security issues in the transportation domain while enhancing the efficiency of traffic prediction and management. In 2017, the classic federated averaging algorithm was introduced. McMahan proposed a federated learning algorithm based on gradients, and the paper discussed how to address data security and privacy issues in a distributed environment, laying an important foundation for the research and application of federated learning [5].

Since 2018, scholars have found that data imbalance and other issues often occur in practical applications. In order to improve the efficiency and scalability of federated learning, Zhao et al. proposed a weighted average based federated learning algorithm [6]. This algorithm adds a weight to the local model updates of each client to balance the impact between different clients. Through experiments on different datasets, the effectiveness and stability of this algorithm have been demonstrated, which can significantly improve the accuracy of federated learning. This paper provides an important reference for federated learning applications that address data imbalance issues.

In 2020, Wei et al. proposed a federated learning algorithm that combines differential privacy protection, aiming to protect the data privacy of participants. This paper introduced a differential privacy protection mechanism based on noise addition, which is applied to model updating and aggregation in federated learning processes [7]. Through experiments on different datasets, this paper proves that the algorithm can protect data privacy while hardly affecting the accuracy of federated learning. This paper provides an effective solution for privacy protection in federated learning.

In 2021, Qi et al. proposed a federated learning algorithm based on differential privacy to improve the accuracy of traffic prediction while protecting data privacy [8]. The article also designed a series of experiments to prove the effectiveness and reliability of the algorithm.

In 2022, Dong et al. proposed a joint meta learning mechanism called padp federa [9] to address the negative impact of non-IID data on predictive accuracy in joint learning. This mechanism can personalize the different privacy costs of each client and has high convergence. Padp federa can also add a certain level of noise to smooth out gradient fluctuations during communication, while personalized client privacy overhead further accelerates convergence speed.

The application of federated learning (FL) in the field of traffic control has increasingly become a focus of attention in both academic and industrial circles in recent years. It offers innovative solutions to address privacy protection, data heterogeneity, and distributed data collaboration issues in traffic control. A paper [10] targeting the speed adjustment problem for connected and autonomous vehicles (CAVs) introduces an intelligent optimization framework that incorporates federated learning. By integrating FL techniques into the speed optimization of CAVs in mixed traffic flow environments, the paper proposes an efficient and privacy-friendly solution. In experiments, this framework significantly improved the learning performance of individual vehicles and overall traffic efficiency, particularly under conditions of moderate traffic demand. It demonstrates the innovative potential of FL in traffic control applications.

### 2.2. Research on Model Lightweighting 

As deep learning models evolve, their computational complexity increases, and the number of model parameters grows, leading to most existing models being overly bulky and redundant. Against this backdrop, methods for model compression and acceleration have been proposed. Among these, model pruning has emerged as one of the most popular and relatively simple yet effective network compression techniques. Model pruning is a technology for reducing the size of deep neural networks by eliminating redundant weights, which can enhance computational efficiency, decrease storage requirements, and speed up the model during both training and inference. The following is an overview of the development of model pruning and a summary of the related literature. Traditional model pruning: Traditional model pruning was proposed by Yann et al. in 1990 in the early stage. This method is called optimal brain damage (OBD) [11]. Its purpose is to compress the network by removing unimportant weights and reduce network overfitting. OBD is a second-order optimization method based on the Hessian matrix, which can calculate the contribution of each weight to the loss function, and then remove some small contribution weights. However, due to the need to calculate the Hessian matrix for OBD, it is difficult to apply it to large-scale deep neural networks.

Method based on structured pruning: The method based on structured pruning is a pruning technique that can organize the weights in a neural network according to certain rules, thereby compressing the neural network. One common method is to use low rank matrix factorization, which can decompose the weight matrix into several small matrices, thereby reducing storage space and accelerating computation. Another common method is to use filter pruning, which can remove unimportant filters, thereby reducing the computational complexity and storage space of the network. For example, Luo et al. proposed the ThiNet method [12] in 2017, which uses a threshold decomposition method to compress the filters in each convolutional layer. This method can reduce the network size by 60% while maintaining accuracy. Distillation pruning method: The distillation pruning method is a method of pruning that utilizes both teacher and student models. This method involves pruning by training a larger teacher model and a smaller student model. The output of the teacher model is used as a label for the student model, thereby reducing the size of the student model while maintaining accuracy. For example, Hinton et al. proposed the knowledge distillation method in 2015 [13], which uses a large model as a teacher model to teach a small model. This method can reduce the model size by 5 to 10 times and achieve similar or better performance on different tasks. Weight pruning method: Weight pruning is an emerging pruning method that can regularly prune weights during the training process, thereby reducing the size and computational complexity of the network. This method can trim after each training iteration and reduce the size of the network without losing accuracy. For example, Franklin and Carbin proposed a one-time pruning method in 2019 [14], which can remove some less important weights during the training process and continue training after pruning, thereby improving the efficiency of the network. Adaptive pruning method is a method that can dynamically prune based on feedback during the training process. This method can continuously remove unnecessary connections during the training process and add new connections as needed, thereby improving the efficiency and accuracy of the network. Unlike traditional pruning methods, adaptive pruning methods can adjust pruning ratios based on specific tasks and datasets, thereby achieving more refined network optimization. For example, Han et al. proposed an adaptive accelerated deep neural network (ADMM) method in 2015 [15], which can remove weights based on the ADMM algorithm while maintaining the accuracy and stability of the network.

In summary, model pruning can improve the computational efficiency and accuracy of deep neural networks and reduce storage requirements.

### 2.3. Personalization of Federated Learning

In federated learning, the “personalization mechanism” plays a key role, especially in distributed training scenarios involving multiple clients. The main goal of personalization is to improve the performance of each client model while protecting data privacy and meeting diverse and specific needs. Federated learning mainly relies on aggregating local model updates from multiple clients to build a globally shared model. However, in traffic flow prediction scenarios, traffic patterns can change significantly in different regions, which can greatly affect data distribution. This data heterogeneity presents challenges, because a single global model may struggle to generalize well across all clients and meet their specific needs. To solve this problem, personalization mechanisms are introduced in federated learning. These mechanisms aim to create personalized models based on the local data characteristics and needs of each client rather than relying only on the global model. By doing this, they not only improve the predictive performance of the local models but also reduce performance decline caused by differences in data distribution among clients. The development of personalized federated learning occurs as follows.

1. Simple personalization: The initial client personalization method uses the client’s feature vectors as input and incorporates them into the global model training process in a simple manner, for example, by adjusting the bias or weight of the model using information such as the client’s language environment and location. Li et al. proposed a novel personalized federated learning algorithm that utilizes the Moreau envelope regularization technique to learn personalized models for each client [16]. This algorithm allows for efficient communication between the clients and the server while preserving individual client privacy. Experimental results on benchmark datasets demonstrate that this method can achieve higher accuracy and faster convergence compared with traditional federated learning algorithms. The proposed algorithm has potential applications in various fields where personalized models are required.
2. Client selective feedback: Client selective feedback is an improved client personalization method that takes feedback from the client as input and selectively integrates feedback information into the training of the global model to improve model accuracy and convergence speed. Zhang et al. proposed a model-agnostic meta-learning (MAML)-based personalized federated learning algorithm that can learn the optimal personalized model for each client with theoretical guarantees [17]. The proposed algorithm can effectively adapt to the differences in data distributions and local models of different clients by using a small number of samples. Theoretical analysis shows that the proposed algorithm can achieve the optimal rate of convergence for the personalized models. Experimental results on benchmark datasets demonstrate the effectiveness of the proposed algorithm in achieving higher accuracy and faster convergence than existing methods.
3. Client caching: Client caching is a method of storing model parameters locally on the client to accelerate global model training. The client trains based on its own feature vectors and local training data, and then uploads the updated model parameters to the server for global model updates. Yang et al. proposed a new federated learning method that utilizes different client caching strategies to improve the personalized performance of the model [18]. This paper uses a weighted average federated average algorithm to update model parameters and improves client performance by personalized adjustments to cached parameters. Through experiments on the MNIST and CIFAR-10 datasets, this method significantly reduces communication traffic and runtime while maintaining accuracy.
4. Client adaptation: Client adaptation is a more advanced method of client personalization. It adapts to the characteristics of the client’s local data by adjusting the client’s hyperparameter, activation function, etc., and improves the generalization ability and prediction performance of the model. Li et al. proposed a federated learning method based on parameter personalization [19]. This method allows different devices to upload different parameters, calculate the similarity of the parameters, and combine it with the global model for personalized training. The experimental results show that this method can significantly improve the performance of personalized devices while ensuring global model performance. In addition, this method can also achieve better performance when the number of devices is small, and has good robustness, which can adapt to the situation of device failure and dynamic addition.

Client personalization in federated learning is a rapidly developing research direction, which is of great significance for improving model prediction performance and efficiency.

At present, some scholars have begun to explore how federated learning is applied in the field of traffic flow forecasting, and have achieved certain results. Liu et al. [20] combined FL with GRU to predict traffic and used the FedAvg algorithm for parameter aggregation to reduce communication costs. The traffic flow prediction results show that the FedGRU algorithm maintains data privacy, although the results of FedGRU are slightly worse than those of other advanced forecasting models. In terms of network-level traffic flow prediction, Mengran Xia et al. combined federated learning with graph convolutional neural networks to predict road network traffic flow, and the prediction results were basically the same as those of other machine learning models. It shows that, while federated learning protects privacy, prediction accuracy can also be guaranteed [21]. Xiaoming Yuan et al. designed a spatiotemporal federated network model FedSTN, and used a VFL-based federated graph attention (FedGAT) layer in the federated learning framework to share parameters. This improves the predictive power of the global model [22]. Another scholar [8] combined blockchain with federated learning and introduced a noise addition mechanism to solve the security problem of the central server in traffic prediction. In addition, Zhang et al. [23] proposed a differential privacy-based adjacency matrix GNN model under the FL framework to protect the privacy of topological information in ITS, and integrated the attention mechanism into the FASTGNN model.

## 3. Methodology

This section will introduce the personalized lightweight federated learning (PLFL) method proposed in this paper, which primarily consists of two components: dynamic model pruning (DMP) and personalized federated learning based on model distance and attention mechanism.

### 3.1. Personalized Lightweight Federated Learning Framework

This paper introduces a personalized lightweight federated learning (PLFL) method for predicting short-term traffic flow. The paper divides different traffic regions into independent and isolated clients, each with its own traffic data that does not interflow with others. Due to the varying quality and quantity of traffic data possessed by each client, the performance of models trained centrally by individual clients is not satisfactory. Federated learning, a distributed learning architecture, can leverage data from all parties to collaboratively train a global model, effectively addressing the shortcomings of insufficient or poor-quality data from individual clients. Moreover, during training, the data does not leave the local clients, ensuring robust data privacy for all parties. With the advancement of deep learning models, their network complexity has increased, and the number of model parameters has grown, leading to most existing models being overly bulky and redundant. In federated learning, the need to transmit model parameters between clients and the server necessitates the reduction of model parameters to alleviate communication burdens. Additionally, in reality, the amount of data available to each client varies, which leads to different influences on the model parameters during local training. For instance, clients with large amounts of data may disproportionately influence the final global model, overshadowing the individuality of other clients and creating an unfair situation. To address this, we propose a personalized federated learning approach based on model distance and attention mechanisms.

### 3.2. Federated Learning Collaborative Training Framework

In the federated learning collaborative training framework, local clients and the server play two key roles, working together to achieve model training and optimization. The local clients serve as the fundamental components of the federated learning system, providing the necessary data resources. Each client can be viewed as an independent working unit, capable of autonomously managing local data and deciding when and how to participate in federated learning. Due to the distributed architecture of federated learning, the dynamic joining or exiting of certain clients does not significantly impact the overall model performance, which offers a high degree of flexibility that is highly valuable to the participants.

In this framework, the primary responsibility of the client is to receive the initial model and its weight parameters dispatched by the server, train the model using local data, and upload the updated model weights back to the server at the end of each training round. The server, functioning as the “central brain” of the federated learning process, operates independently of the data owners of all clients and does not directly access any local data. Its main responsibilities include initializing the global model and parameters, distributing them to the clients, receiving and aggregating the model parameters uploaded by the clients, generating a new global model, and redistributing it to the clients for the next iteration. Throughout this process, the server coordinates and manages operations while ensuring that data privacy is upheld, which is a significant advantage of federated learning.

**Figure 1:**
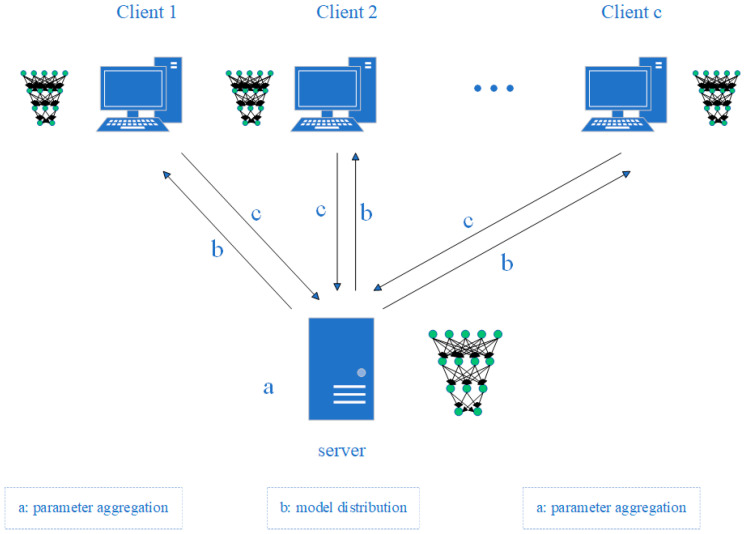
Collaborative training process of federated learning.

The collaborative training process of federated learning is shown in Figure 1. The specific steps are as follows.

1. Pre-train the initial global model on the server side to obtain the initial weight parameters of the global model.
2. Distribute the initial weight parameters of the server-side global model to each client through the communication network.
3. The client receives the initial weight parameters of the global model sent by the server and iteratively trains the model using local data.
4. The client uploads the weight parameters of the local model completed in this round of training to the server through the communication network.
5. The server aggregates the weight parameters of the model transmitted by various clients to obtain a new global model.
6. Continue with the next round of communication and repeat steps (2)–(5) to obtain the final prediction model after reaching the set number of communication rounds.

### 3.3. Local Model

The spatial traffic network is often defined as an undirected graph M = (S, W, A), where S is the set of all stations, W represents the set of edges, *N* represents the number of nodes, i.e., *N* = | S |, and the *i* th traffic station is represented by S*i*. *A*∈R*N* ×*N* is the adjacency matrix of the spatial transportation network, which can directly represent the adjacency relationship between stations.

The task of traffic flow prediction is simply to predict the future traffic flow value of several stations based on their historical traffic flow values. The traffic flow value observed on the spatial traffic network M at the nth time step is defined as the graph signal matrix f(t) ∈ R*N* × *F*, where *F* refers to the number of features collected at each station. The input graph signal matrix is defined as f = (f (*t* − *T* + 1), f (*t* – *T* + 2), ⋯⋯, f (*t*)) ∈ R*T* × *N* × *F* composed of *T* historical time steps of traffic flow observations. The graph signal matrix to be predicted is g = *T* × *N* × *F* as g = (f(*t* + 1), f(*t* + 2), ⋯⋯, f(*t* + V) ∈ RV × *N* × *F*, composed of V future time steps of traffic flow values. The goal of the traffic flow prediction task is to learn a function mapping relationship, as shown in the equation:

(1) $$\left[f,M\right]\overset{y\left(.\right)}{\to }g$$

This paper employs a multi-head self-attention time convolutional network (TCN) to extract temporal features from traffic flow data. Initially, a multi-head self-attention model is utilized to pre-process the time series data, enabling the learning of features from different subspaces and obtaining richer potential information. Subsequently, TCN is employed to extract temporal features from the processed time series data and capture the dependencies between local and remote time [24].

In a single-head self-attention model, assuming there are T historical traffic flow parameters, N urban road nodes, and each urban road node has F features, given the model input f = (f (*t* − *T* + 1), f (*t* – *T* + 2), ⋯⋯, f (*t*)), the single-head self-attention model can obtain three subspaces, Q, K, and V, based on the same input f. The learning process of these three subspaces can be expressed as formulas (2)–(4).

(2) $$Q=fW^{Q}$$

(3) $$K=fW^{K}$$

(4) $$V=fW^{V}$$

Scale dot product attention is a calculation method of attention mechanism, whose dot product is reduced according to $\sqrt{d_{K}}$. The formula for calculating attention using the dot product of scales is:

(5) $$Attention\left(Q,K,V\right)=softmax\left(\frac{QK^{T}}{\sqrt{d_{K}}}\right)V$$

where $\sqrt{d_{K}}$ represents the dimension of the key.

The multi-head self-attention model is equivalent to merging multiple single-head self-attention models, where each single-head structure is only responsible for outputting one subspace in the sequence, and finally merging the calculation results of each subspace to obtain the final output sequence. If the model has W heads, then one self-attention structure is responsible for 1/N of the output. The more heads there are, the more abundant potential information can be obtained, but the computational complexity will also increase. This paper uses a multi-head self-attention model with two heads to process input data.

(6) $$Multihead\left(Q,K,V\right)=Connect\left({head}_{1},\ldots ,{head}_{h}\right)W^{C}$$

(7) $${head}_{i}=Attention\left(Q_{i}.K_{i}.V_{i}\right)$$

(8) $$Q_{i}=fW_{i}^{Q}$$

(9) $$K_{i}=fW_{i}^{K}$$

(10) $$V_{i}=fW_{i}^{V}$$

After the data are processed by a multi-head self-attention model, residual connections are used to standardize the results layer by layer, and the standardized results are input into TCN. Given that the dilated convolution in TCN adopts a zero padding strategy to maintain the length of the time series constant, and then uses the same convolution kernel for all elements in the input sequence, the calculation formula for TCN is as follows.

(11) $$Y_{Tcn}=\theta {*}_{d}f$$

where *_d_ is the expansion convolution, d is the expansion rate, and θ is the temporal convolution kernel. So, at time t, the formula for calculating the TCN result $y_{i,t,p}$ of the road node V_i_ on the p-th channel is as follows.

(12) $$y_{i,t,p}=\sum_{\alpha =1}^{A_{\tau }}\sum_{z=1}^{Z}\theta_{\alpha ,z,p}.f_{i,t-d\left(\alpha -1\right),z}$$

where d is the dilation rate of the dilated convolution in TCN, $\theta_{\alpha ,z,p}$ forms the time convolution kernel θ∈R^(A_τ_ × Z × P^*), A_τ_ represents the kernel length, and P^* represents the number of output channels.

The output of the multi-head self-attention time convolutional network in the l-th layer is $Y_{Tcn}^{l}$, calculated as follows.

(13) $$Y_{Tcn}^{l}=\left\{\begin{array}{cc} f, & l<0\\ \delta (\theta^{l}{*}_{d^{l}}Y_{Tcn}^{l-1}), & l\geq 0 \end{array}\right.$$

If l = 0 represents the input layer, the network takes the output of the multi-head self-attention model as input, where $\theta^{l}$ represents the dilated convolution kernel of TCN, and δ(.) denotes a nonlinear function.

Urban traffic conditions are constantly changing, and the correlation between roads also dynamically changes with changes in traffic conditions. This means that the weight of the correlation between roads should also be dynamically changing. In response to the above issues, this paper proposes GAT-GCN, which is jointly implemented by GAT and GCN.

Using a GAT to calculate the attention scores for the relationships between road nodes, it dynamically weights the spatial neighbor subgraphs at different times, enabling them to adapt dynamically according to the actual traffic situation of the road network. The biggest feature of the attention mechanism is that it allows the model to have different inputs, focusing on the input that is most relevant to the output and making decisions based on it. After obtaining dynamically weighted spatial neighbor subgraphs, GCN is used to extract spatial correlations from the dynamically weighted subgraphs, and the spatial characteristics of urban traffic flow are comprehensively described. GAT can obtain the importance level of all neighboring nodes of each node by processing and calculating each node in the graph. Then, weights are assigned to neighboring nodes based on their importance. Nodes that have a significant impact on the results are assigned more weights, while nodes with smaller weights are assigned to make the model pay more attention to important nodes in the graph.

In multi-head graph networks, it is important to note that the input to the network layer is a set of node features. In the case of an N-node road network, where the feature vector of each road node is $h_{i}$ and has a dimension of D:

(14) $$h=\left\{h_{1},h_{2},\ldots ,h_{N}\right\},h_{i}\in R^{D}$$

The $h_{i}$ of each node is linearly transformed to obtain a new feature vector $h_{i}^{\prime }$ with dimension $D^{\prime }$. As shown below, where $W\in R^{D\times D^{\prime }}$ is the weight matrix of the linear transformation:

(15) $$h_{i}^{\prime }=Wh_{i},\;W\in R^{D\times D^{\prime }}$$

(16) $$h_{i}^{\prime }=\left\{h_{1}^{\prime },h_{2}^{\prime },\ldots ,h_{N}^{\prime }\right\},h_{i}^{\prime }\in R^{D^{\prime }}$$

Assuming that node $V_{i}$ is a neighbor of node $V_{i}$, we can use the shared attention mechanism to calculate the attention coefficient between these two adjacent nodes in the graph. The attention coefficient between nodes *V_i_* and *V_j_* can be calculated as follows.

(17) $$e_{ij}=Attention\left(Wh_{i},Wh_{j}\right)$$

The specific approach of the attention mechanism is to concatenate the feature vectors *h_i_* and *h_j_* of adjacent nodes $V_{i}$ and $V_{i}$ together, and then calculate the inner product with an $R^{D^{\prime }}$×$R^{D^{\prime }}$→R-dimensional vector a. If LeakyRelu is used as the activation function, the calculation formula for attention is as follows.

(18) $$e_{ij}=LeakyRelu(\alpha^{T}\left[Wh_{i}|\left|Wh_{j}\right]\right)$$

In order to make the attention coefficients easy to compare between different nodes, it is necessary to standardize the attention coefficients. The normalization method is used for processing.

(19) $$\alpha_{ij}=softmax\left(e_{ij}\right)=\frac{\exp\left(e_{ij}\right)}{\sum_{Z\in U_{i}}\exp\left(e_{iZ}\right)}$$

where $U_{i}$ represents a set of neighboring nodes of node $V_{i}$.

The dynamic attention coefficient *α_ij_* can be obtained through the above formula, and all $\alpha_{ij}$ form the dynamic attention coefficient matrix $C\in R^{D\times D}$. The dynamic attention coefficient between nodes in the spatial neighbor subgraph is calculated, where $C_{sp}$ represents the dynamic attention coefficient matrix of the spatial neighbor subgraph. The calculation formula for the dynamic road weight Z of spatial correlation is as follows.

(20) $$Z=\tilde{A}⊙C$$

where ${A=A}_{sp}+I$, where I is the identity matrix. ${C=C}_{sp}$. The dynamic road weight matrix corresponding to the spatial neighbor subgraph is represented as $Z_{sp}$.

After attention calculation, the eigenvector calculation formula for node $V_{i}$ is as follows.

(21) $$h_{i}^{\prime }=\sigma \left(\sum_{j\in U_{i}}\alpha_{ij}Wh_{j}\right)$$

To obtain richer features between nodes, the model utilizes a multi-head graph attention network to enhance its learning ability. Assuming that the multi-head graph attention network comprises K attention heads, each attention head is used to perform transformations separately. The vectors generated by the K attention heads are then concatenated together to obtain the final result. The formula for calculating the eigenvector of node $V_{i}$ is as follows.

(22) $$h_{i}^{\prime }=Connect\left[\sigma \left(\sum_{j\in U_{i}}{\alpha_{ij}}^{1}W^{1}h_{j}\right),\ldots ,\sigma \left(\sum_{j\in U_{i}}{\alpha_{ij}}^{k}W^{k}h_{j}\right)\right]$$

where Connect represents serial connection.

Formula (22) represents the feature vector output when a non-last node of the multi-head graph attention mechanism is used, while the output for the last node of the multi-head graph attention mechanism is obtained using the averaging method.

(23) $$h_{i}^{\prime }=\sigma (\frac{1}{K}\sum_{k=1}^{K}\sum_{j\in U_{i}}{\alpha_{ij}}^{k}W^{k}h_{j})$$

By using a multi-head graph attention network, a new feature matrix $h_{i}^{\prime }$ is obtained, and the feature matrix $H=h_{i}^{\prime }$ is updated. At the same time, the dynamic road weight matrix Z is obtained from formula (21), and the feature matrix H and dynamic road weight Z are input into the graph convolutional neural network GCN model to extract the spatial correlation of subgraphs. The interlayer transfer process of GCN is as follows.

(24) $$Y^{l}=\left\{\begin{array}{c} H,\;\;\;\;\;\;\;\;\;\;\;l=0\\ \sigma \left(ZY^{\left(l-1\right)}W^{l}\right),l=1,2,\ldots ,L \end{array}\right.$$

where $Y^{l}$ represents the output of layer l, and if l = 0 is the input layer, then Y is the feature matrix H. $W^{l}$ represents the learnable weight matrix of layer l, and σ(.) is the non-linear activation function representing a non-linear model. This paper chooses a two-layer GCN model to extract spatial correlation.

(25) $$GCN\left(H,Z\right)=\sigma \left(ZRelu\left(ZHW_{0}\right)W_{1}\right)$$

where H is the feature matrix, Z is the dynamic road weight, $W_{0}$ and $W_{1}$ represent the learnable weight matrices between layers in the model, and Relu() represents the corrected linear unit.

Using GCN to extract spatial correlation from the spatial neighbor subgraph $G_{GCN}=(V,E_{GCN})$, formula (25) yields.

(26) $$Y_{GCN}=GCN\left(H_{GCN},Z_{GCN}\right)=\sigma \left(Z_{GCN}Relu\left(Z_{GCN}H_{GCN}W_{0}\right)W_{1}\right)$$

where $Y_{GCN}$, $H_{GCN}$, and $Z_{GCN}$, respectively, represent the spatial correlation, feature matrix, and dynamic road weight matrix of the spatial neighbor subgraph.

After obtaining the high-dimensional features $Y_{TCN}$ and $Y_{GCN}$ in the time and space domains, operations are performed to change the shape of the data for each of them. The data shapes of $Y_{TCN}$ and $Y_{GCN}$ are changed to be consistent in order to aggregate spatiotemporal information for multi-step prediction in the future. Assuming that ${Y^{Tcn}}_{i}$ and ${Y^{GCN}}_{i}$ represent the high-dimensional temporal and spatial features of the node $V_{i}$ to be aggregated, respectively. This paper chooses to use the optimal weight method to fuse the high-dimensional temporal and spatial features obtained from the time prediction algorithm MH-TCN and the spatial prediction algorithm MG-GCN in order to obtain high-dimensional spatiotemporal features. The optimal weight method is to calculate the variance of the prediction results of all prediction models within the combination, and use the minimum variance criterion to determine the weight of each prediction model. Assuming $W_{Tcn}$ and $W_{GCN}$ are the weights of high-dimensional temporal and spatial features, respectively, the fused high-dimensional spatiotemporal features can be represented as:

(27) $$Y_{i}=W_{Tcn}.{Y^{Tcn}}_{i}+W_{GCN}.{Y^{GCN}}_{i}$$

After obtaining high-dimensional spatiotemporal features, the cumulative prediction method for traffic flow prediction is chosen. Specifically, the cumulative prediction method adds the current prediction result to the input for the next prediction step. At this point, the prediction error values for each step are also accumulated. For long-term prediction, the accumulation of error values will continue to rise, causing significant errors. The model in this article adopts the method of directly predicting the future M steps to avoid the accumulation of errors caused by inaccurate predictions in the early stage.

**Figure 2:**
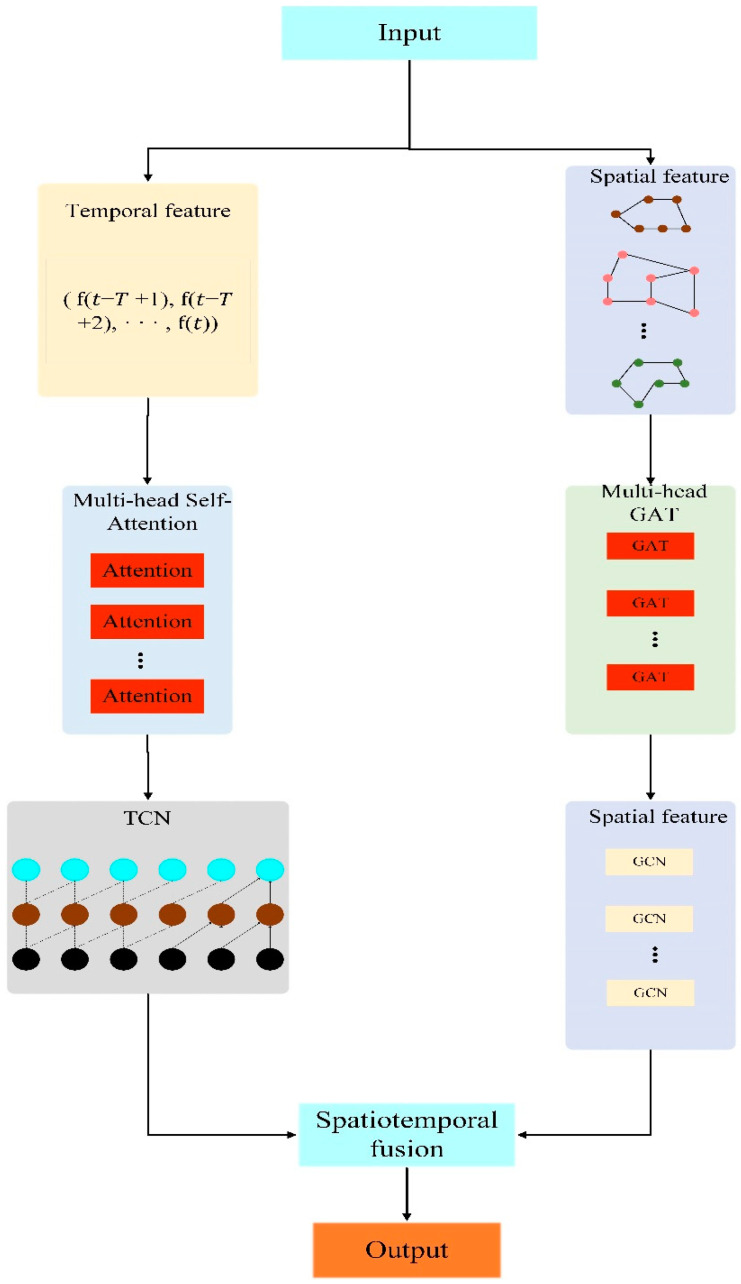
Spatiotemporal fusion graph convolution model (MGTGCN).

The specific framework of the spatiotemporal fusion graph convolution model (MGTGCN) is shown in Figure 2.

### 3.4. Model Pruning

The iterative algorithm running in federated learning requires low latency and high throughput connections between computing units. However, aggregation servers typically require a large number of edge nodes or terminal devices to be connected through a resource-limited spectrum. Therefore, in the global aggregation step of federated learning, only a limited number of edge nodes or terminal devices are allowed to send trained and updated model parameters through unreliable channels. In addition, the computing capabilities of edge nodes and terminal devices participating in federated learning vary, and in the process of local computing, the amount of computation should be controlled within the range of device computing resources and capabilities. Under this limitation, pruning and compressing deep learning models has become an effective method.

The most commonly used method for model pruning is the three step method: pre-training, pruning, and fine-tuning. Firstly, a large highly redundant model is trained, and then the redundant weights are pruned according to certain criteria during the pruning process while retaining important weights. Finally, the weights inherited by the pruning model are fine-tuned to best maintain accuracy before pruning. This means that the weight after pruning is crucial to the performance of the model.

However, recent studies have shown that pruned models can be trained from scratch without fine-tuning the weights obtained from the complete model training, and can also achieve considerable predictive performance. However, fine-tuning the pruning model will only result in an equivalent or worse performance than training it with random initial weights, indicating that the pruned structure is more important for the performance of the pruned model compared with inherited weights. Therefore, this article adopts a dynamic pruning strategy of retraining the model after each pruning to improve the accuracy of the model. During the training process after pruning, if the accuracy decreases significantly, the threshold will be increased. If the accuracy remains unchanged or the decrease is minimal, the threshold will be lowered in the next round of pruning.

**Figure 3:**
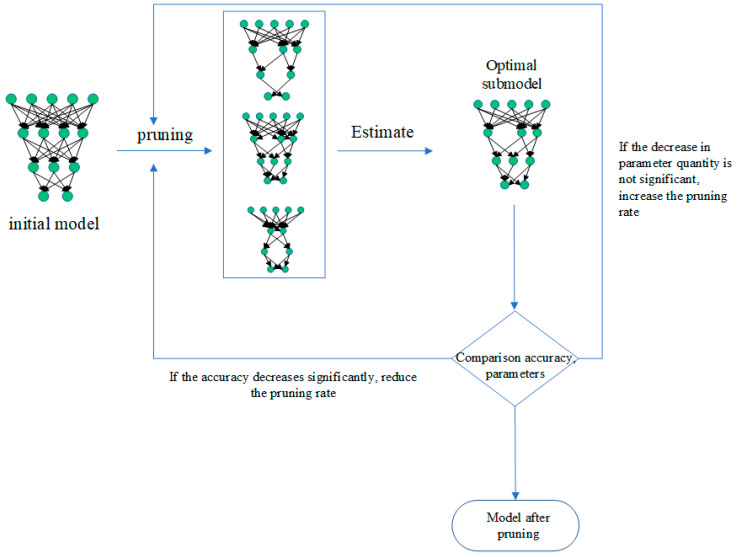
Dynamic model pruning process.

The specific operation process is shown in Figure 3.

### 3.5. Client Personalization Mechanism

In order to address the issue of imbalanced samples among various clients in federated learning, large sample clients with large balance weight parameters have a significant impact on the final model training results, ensuring the personalization of each client. This paper incorporates an attention mechanism between the client and server to participate in the parameter aggregation of the global model. The attention mechanism is designed to provide each client model with appropriate attention weights. These weight values correspond to the model parameters at each layer of the neural network. By using these attention weights as aggregation coefficients, it is possible to enhance the global model’s ability to capture critical information about features.

The formula symbols in this section are explained in the following Table 1.

The attention mechanism introduced in this paper aims to minimize the weighted distance between the client and server during communication. Continuously updating parameters during communication iterations, this mechanism can fully utilize the relationship weights at both ends of the communication. And this mechanism can also improve the performance of the final global model by extracting the importance of each layer of neural network from each client model. When the server aggregates, it calculates the distance between the current local model parameters and the pre-processed global model parameters to determine the differences between the models. Thus, personalized weights are developed for the local model, and then the weighted average is used to obtain the global model. This approach can maintain the personalized characteristics of each model based on mutual learning among models. The formula is as follows:

(28) $$F\left(W_{n}^{s},W_{n+1}^{1},\ldots ,W_{n+1}^{C}\right)=argmin\sum_{c=1}^{C}\left[\frac{1}{2}A_{c}D{\left(W_{n}^{s},W_{n+1}^{C}\right)}^{2}\right]$$

(29) $$z=\sum_{c=1}^{C}A_{c}(W_{n}^{s},W_{n+1}^{C})]$$

In the feature attention mechanism section, we obtain the importance weight parameters of each layer of neural network at n times for each client model using formulas (28) and (29). Combined with the weight parameters of the global model at time n, the global model parameters are updated at time n+1 through Formula (30):

(30) $$W_{n+1}^{s}=W_{n}^{s}-\alpha z$$

In Formula (31), we use layered soft attention to calculate the value of A. Specifically, we view the client model as a key, and, correspondingly, we view the global model as a query. On this basis, we calculate the attention scores of each layer of the network.

(31) $$A_{c}^{l}=softmax\left({\left\|W^{l}-\right.\left.W_{c}^{l}\right\|}_{p}\right)$$

**Figure 4:**
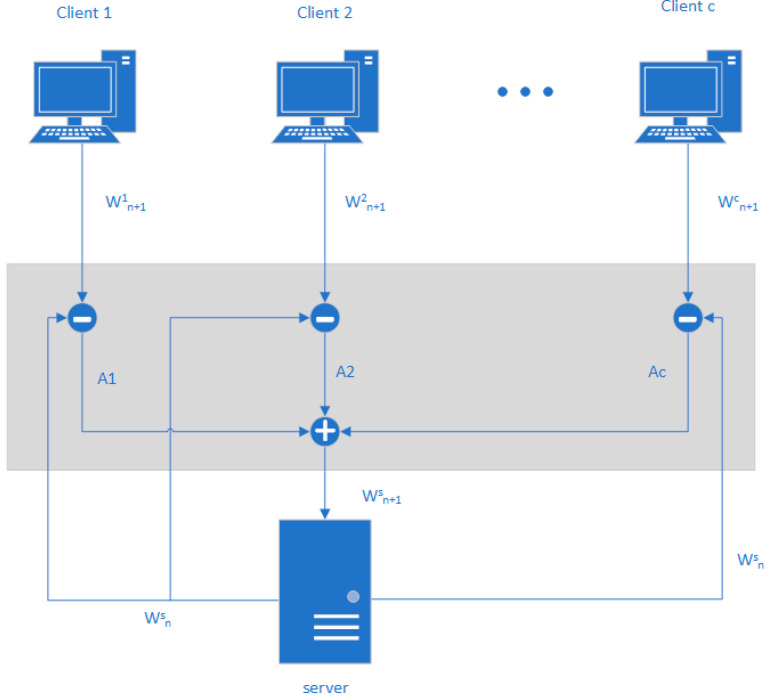
Client personalization mechanism.

The specific operation process is shown in Figure 4.

The computational complexity of the client personalization mechanism primarily consists of calculating the parameter distance between the global model and the local models, along with the computation of hierarchical attention weights. For each client c, it is necessary to compute the Euclidean distance D between the parameters of the local model $W_{n+1}^{C}$ and the global model parameters $W_{n}^{S}$, with the formula as follows:

(32) $$D\left(W_{n}^{s},W_{n+1}^{C}\right)={\left\|W^{l}-\right.\left.W_{c}^{l}\right\|}_{2}$$

Assuming that the number of parameters in each model is P, the complexity of calculating the Euclidean distance is O(P), as it requires calculating the squared difference between each parameter and then summing them up. For C clients, the total complexity for distance calculation is:

(33) $$O\left(C,P\right)$$

The computation of hierarchical attention weights follows the formula:

(34) $$\left(O\left(C,L,\bar{P}\right)\right)$$

where L is the number of layers in the model, and $\bar{P}$ is the parameter dimension per layer. Thus, the computational complexity of the client personalization mechanism is:

(35) $$O\left(C,P\right)+O\left(C,L,\bar{P}\right)$$

It can be observed that an increase in C significantly raises the computational complexity. Therefore, in scenarios with a large number of clients, this mechanism may incur considerable computational costs. The use of this mechanism should be targeted at scenarios with a moderate number of clients (such as hundreds to thousands), where there is a high demand for personalized weights and communication aggregation effects, such as in personalized recommendation systems and regional traffic flow predictions.

### 3.6. Model Aggregation

The server, when aggregating, needs to calculate the distance between the current local model parameters and the pre-processed global model parameters to determine the dissimilarity between models. This allows for the creation of personalized weights for the local models, which are then used to compute a weighted average to obtain the global model. This approach maintains the individual characteristics of each model while enabling mutual learning among them.

The global model employs a federated aggregation algorithm based on a feature attention mechanism, which provides appropriate attention weights for each local model. These weights correspond to the model parameters at each layer of the neural network. It is important to note that, when local model parameters are uploaded to the server, there may be a weight mismatch issue when calculating the attention scores for each layer in conjunction with the global model from the last communication. Therefore, before calculating the attention scores for each layer, the weights of the last fully connected layer in all client models need to be averaged. This average is then assigned to the last layer parameters of the global model from the last communication. The calculated attention weights are used as aggregation coefficients to enhance the global model’s ability to capture critical feature information.

## 4. Data Description

This section primarily focuses on introducing the traffic flow dataset used in the experiments conducted for this paper.

We select license plate recognition (LPR) data from Changsha city as the data source for the experiment. The data are collected through monitoring devices installed in the urban road network, covering vehicle passage records from 1 September to 30 September 2019. This dataset accurately reflects the dynamic characteristics of traffic flow in Changsha during that period. The original LPR data consist of eight fields: license plate number, license plate type, passage time, collection point number, collection point name, equipment number, direction number, and lane number. The license plate number is used to identify each vehicle uniquely, while the license plate type reflects the distribution of different types of vehicles across the road network. The passage time records the exact time a vehicle passes the monitoring point, and the collection point number and name identify and describe the geographical location of the monitoring points, respectively. The equipment number indicates the uniqueness of the collection device, and the direction and lane numbers record the vehicle’s driving direction and lane position. To improve the usability of the data and the efficiency of the experiment, we pre-process the original data. First, we clean the dataset by removing duplicate records and anomalies (such as missing fields or incorrect time formats). Then, we aggregate the data into five-minute intervals to obtain traffic flow data. We process the data to obtain traffic data in five minutes, and 80% of the dataset is the training set and 20% is the testing set.

**Figure 5:**
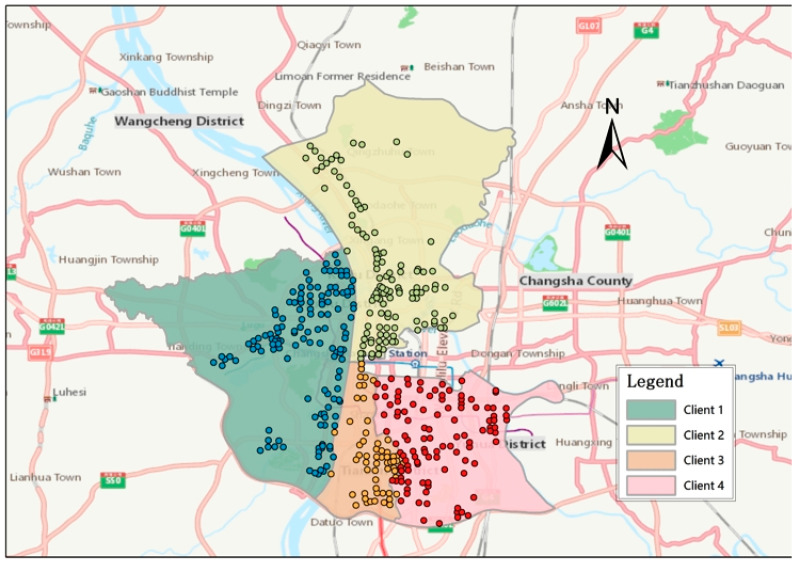
Data description.

In Figure 5, four administrative districts of Changsha are selected by the blue border: Yuelu District, Kaifu District, Tianxin District, and Yuhua District. We set each zone as an independent client, i.e., Client 1, Client 2, Client 3, and Client 4.

## 5. Experimental Analysis

### 5.1. Experimental Setup and Evaluation Function

All experiments are conducted on the AMD Ryzen 7 5800H processor, NVIDIA GeForce RTX 3070 graphics card, 16GB memory, and Python 3.9.

We set four LPR data collection manufacturers in Changsha as four clients and select one month’s traffic flow data for each manufacturer’s road section for experiments. The number of communication rounds for federated learning is set to 50.

The model training employes the following configuration.

Hyperparameters: learning rate = 0.001, batch size = 32, number of training epochs = 50.

Optimization algorithm: the Adam optimizer, which accelerates gradient descent and improves the convergence speed of the model.

Loss function: mean squared error (MSE), used to measure the difference between predicted and actual values.

Federated learning settings: Each client independently trains a local model and uploads model weights to the server at the end of each training epoch. The server aggregates the model weights from different clients to update the global model. This process is iterated until the model converges or reaches the predefined number of training epochs.

RMSE (root mean square error) and MAE (mean absolute error) are commonly used evaluation functions in traffic flow prediction, used to measure the error size between the model’s predicted value and the actual value. Their calculation formula is as follows.

(36) $$RMSE=sqrt\left(\frac{1}{y}*\Sigma {\left(Q_{i}-{\hat{Q}}_{i}\right)}^{2}\right)$$

(37) $$MAE=\frac{1}{y}*\Sigma \left|Q_{i}-{\hat{Q}}_{i}\right|$$

where y represents the number of samples in the dataset, *Q_i_* represents the actual value of the i-th sample, and ${\hat{Q}}_{i}$ represents the predicted value of the i-th sample. The smaller their values, the better the prediction performance of the model.

### 5.2. Model Pruning Experiment

**Figure 6:**
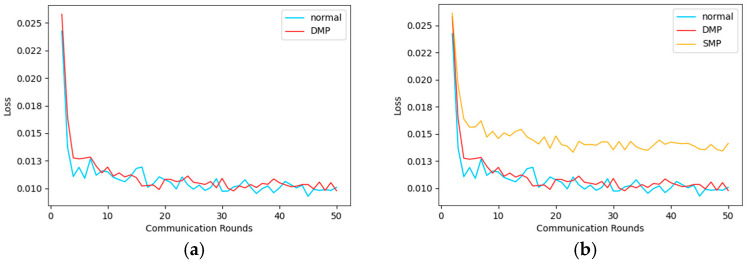
Comparison of results between SMP and DMP. (**a**) Comparison of DMP and Normal Model Loss Values. (**b**) Comparison of DMP and SMP Model Loss Value.

Firstly, we select Client 3 as the research object. Then, we conduct static model pruning (SMP) and dynamic model pruning (DMP) on the client model, respectively. The specific comparison is shown in Figure 6. Among them, the horizontal axis represents the number of communication rounds, and the vertical axis represents the loss value. Here, we use mean in Python’s sklearn. metrics_ squared_ error. A is the comparison between the model loss value under non-pruning conditions and the model loss value after using DMP, while B is the comparison with the model loss value after adding SMP.

From Figure 6, we can see that, after pruning the model with DMP, there is no significant loss in the accuracy of the model, and, compared with SMP, the model with pruning performs relatively poorly. Because static model pruning is only based on a set single threshold, it may damage the integrity of the model structure during the pruning process. Although both dynamic and static model pruning improve the efficiency of the model by reducing the number of model parameters, dynamic model pruning has more flexible, efficient, and adaptive characteristics compared with static model pruning. And because DMP adopts a dynamic pruning strategy of retraining the model after each pruning, it improves the accuracy of the final model. And dynamic model pruning can preserve the integrity of the model structure. This can avoid potential over-pruning issues in static model pruning and reduce the difficulty of debugging and optimization.

Table 2 shows the time spent on federated communication before and after model pruning, indicating that after pruning the model, the federated communication time is significantly reduced. Although the communication time after using SMP pruning is shorter, the accuracy loss of the corresponding model is also significant. Overall comparison shows that DMP is superior. In addition, we also used model parameter values, FLOPs, and model prediction accuracy to represent the results of the dynamic pruning method DMP proposed in this paper. The detailed information of the relevant results can be found in Appendix A.

### 5.3. Personalized Experiment

In order to address the issue of imbalanced samples among various clients in federated learning, the excessive impact of large sample clients on the final model training results is balanced, and the personalization of each client is ensured. We add an attention mechanism between the client and server to participate in parameter aggregation of the global model. We select four clients as experimental subjects. Client 4 is set as the large sample data size (one month) and Clients 1,2, and 3 as the small sample size (one week). We compare the predictive performance of the four clients before and after adding personalized mechanisms, and the results are presented in a box chart.

**Figure 7:**
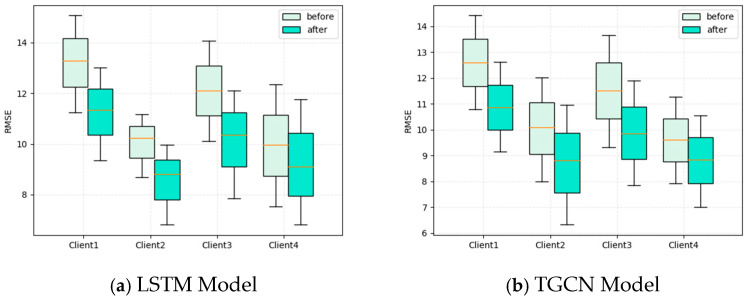

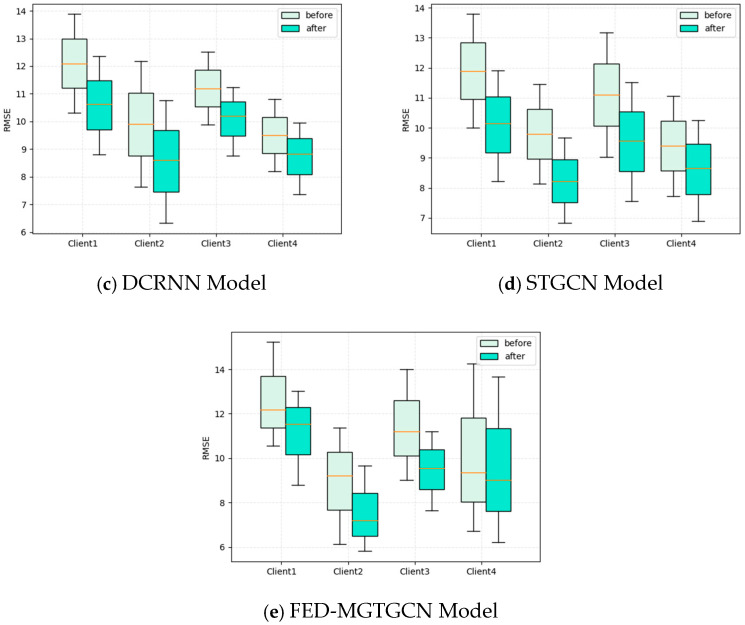
Prediction performance of four clients before and after adding personalization mechanism.

From Figure 7, we can see that the prediction performance of Client 1, Client 2, and Client 3 is relatively poor before adding personalization mechanisms. However, Client 4 performs better. We infer that this is due to the significant impact of Client 4 with a large sample size during the aggregation of global model parameters, resulting in the global model leaning towards Client 4. Therefore, unfair prediction results are caused to the other three clients. After adding a personalization mechanism, the differences between the current local model parameters and the pre-processed global model parameters are calculated to determine the personalized weights for the local model. Then, the weighted average is used to obtain the new global model. The new global model maintains the personalized characteristics of each client model based on mutual learning between client models. From Figure 7, we can also see that, after adding the personalization mechanism, the model prediction performance of Client 1, Client 2, and Client 3 is improved to a certain extent. At the same time, the model prediction performance of Client 4 does not sacrifice much. This indicates that, after incorporating personalized mechanisms, federated learning not only fully utilizes the data of each client to complete the training of the global model but also maintains the uniqueness of each client itself, achieving a “win–win” effect between various clients.

### 5.4. Traffic Flow Prediction Experiment Under Personalized Lightweight Federated Learning Framework

**Figure 8:**
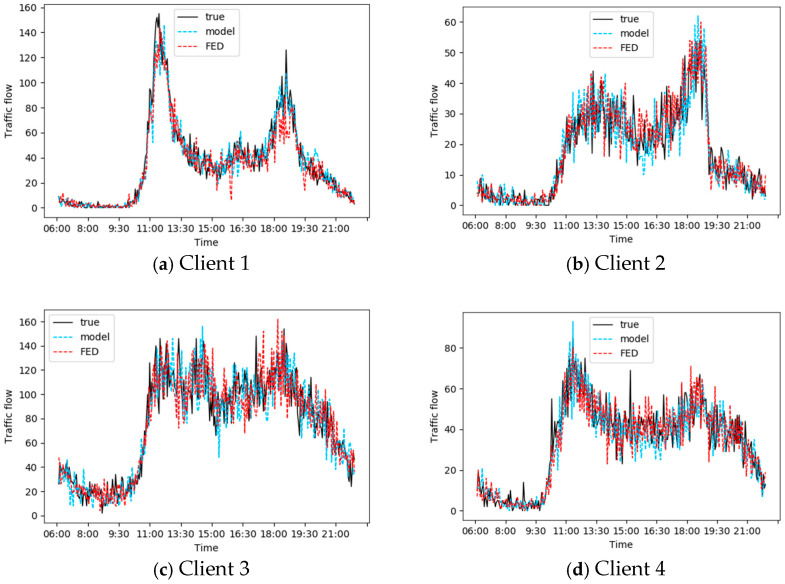
Prediction performance of 4 clients.

We set the number of communication rounds of federated learning to 50. Figure 8 shows the traffic flow prediction results of four clients under the personalized lightweight federated learning framework and the comparison of the results under the traditional centralized model training framework.

It can be seen that the prediction performance of the personalized lightweight federated learning framework proposed in this paper can roughly match that of the centralized training model on various clients.

**Figure 9:**
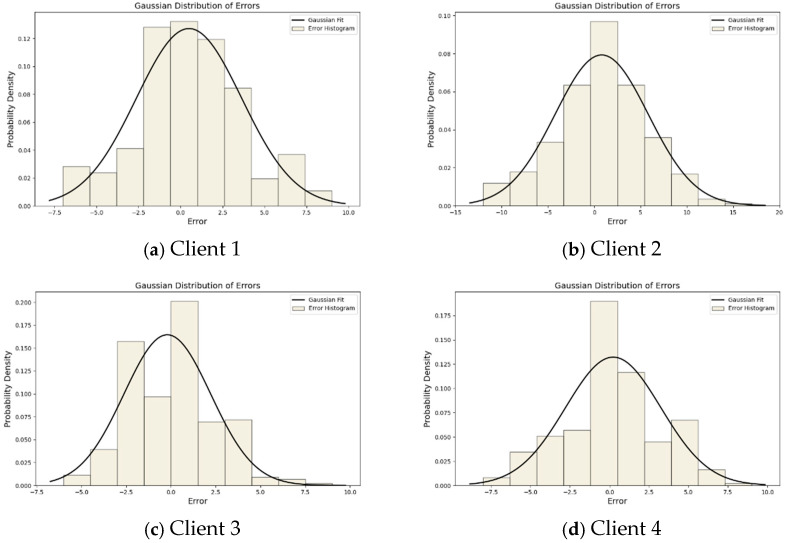
Gaussian distribution of 4 clients.

In order to assess and analyze the accuracy and distribution characteristics of the PLFL model predictions more intuitively, we present the predictions of four clients in a statistical manner. Figure 9 shows the Gaussian distribution plots of the four clients.

From Figure 9, it can be observed that the Gaussian distribution plots of the PLFL model on the four clients display a relatively accurate distribution of prediction errors with typical Gaussian characteristics. These plots reveal that the majority of prediction errors across different clients are concentrated around the mean and gradually decrease towards the sides, forming a bell-shaped curve, indicating the model’s high prediction accuracy and consistency.

In the Gaussian distribution plot of Client 1, the error distribution exhibits a closely packed normal distribution shape, with the mean close to zero and a small standard deviation, reflecting minimal and concentrated prediction errors on this client, with negligible systematic bias. Similarly, the error distribution plot for Client 2 also displays a similar normal distribution shape, with errors concentrated around zero, indicating reliable model performance on this client. Client 3’s Gaussian distribution plot continues this trend, presenting a uniform bell-shaped curve, demonstrating good model performance in handling this client’s data. The error distribution plot for Client 4 shows a slightly wider bell-shaped curve, but with the mean still close to zero and a moderate standard deviation, indicating that the model effectively captures the data characteristics of this client, with overall small prediction errors.

To comprehensively evaluate the performance of the PLFL model, we compare its predictive performance with several baseline models:

- LSTM (long short-term memory network): LSTM is a special type of recurrent neural network (RNN) designed specifically to address sequence learning problems [25].
- TGCN (temporal graph convolutional network): TGCN combines graph convolutional networks (GCNs) and gated recurrent units (GRUs) for modeling spatiotemporal data. TGCN achieves efficient learning and prediction of spatiotemporal data by performing convolution operations on graph-structured data, making it suitable for tasks like traffic flow prediction [26].
- DCRNN (diffusion convolutional recurrent neural network): DCRNN is a model that combines diffusion convolution with recurrent neural networks, focusing on spatiotemporal sequence prediction. By simulating the information diffusion process on a graph, DCRNN can more accurately predict spatiotemporal data, making it especially useful for traffic flow prediction and sensor network data analysis [27].
- STGCN (spatiotemporal graph convolutional network): STGCN is a model that combines graph convolutional networks and one-dimensional convolutional neural networks (1D-CNN). It jointly learns spatial and temporal features to efficiently model and predict spatiotemporal data, applicable to fields such as traffic and meteorology [28].

This paper highlights the significant advantages of the personalized lightweight Federated learning (PLFL) framework by comparing the performance of different models in short-term traffic flow prediction. As indicated in Table 3, PLFL demonstrates substantial superiority over other mainstream models, such as LSTM, TGCN, DCRNN, and STGCN, in terms of prediction accuracy (measured by RMSE and MAE). Specifically, PLFL achieves RMSE values of 7.11, 7.63, and 8.21, and MAE values of 4.05, 4.79, and 4.98 for 5-min, 15-min, and 30-min predictions, respectively, showcasing the best prediction performance. Particularly in short-term (5-min) predictions, the accuracy improvement is especially notable.

This suggests that PLFL is capable of capturing the dynamic trends of traffic flow more accurately, making it suitable for scenarios requiring high-precision short-term predictions, such as intelligent traffic management and control. Additionally, even in longer-term (30-min) predictions, PLFL maintains a low error rate, demonstrating its strong robustness in modeling long-range spatiotemporal dependencies. The superior performance of PLFL comes from various innovative advantages. Firstly, personalized federated learning methods solve the fairness issue caused by differences in client data volume in traditional federated learning by assigning personalized weights. At the same time, the personalized characteristics of each client are retained during global parameter aggregation, making the model more adaptable to diverse traffic scenarios. Secondly, PLFL has designed a spatiotemporal fusion model that combines MH-TCN, GAT, and GCN. MH-TCN extracts local and long-range dependencies of time series, GAT dynamically captures key spatial adjacency relationships in transportation networks, and GCN further extracts deep spatial correlations to comprehensively model complex spatiotemporal features. Thirdly, the dynamic model pruning strategy reduces the number of uploaded model parameters, significantly reduces communication overhead, and enhances practicality in resource constrained environments. Therefore, PLFL not only significantly improves the accuracy of short-term traffic flow prediction but also demonstrates strong comprehensive capabilities in personalized modeling, efficient communication, and spatiotemporal feature fusion, with the potential for widespread application in intelligent transportation systems and urban planning.

### 5.5. Comparison of Prediction Performance of Different Personalized Federated Learning Methods on Different Clients

To investigate the prediction performance of different personalized federated learning methods on various client devices, we conduct comparative experiments between our proposed PLFL method and several mainstream personalized federated learning approaches.

FedTC [29]: This is a personalized federated learning method with two classifiers that preserves personalized information within the local model and enhances the model’s performance on local data. It allows for the sharing of all layers of the local model, ensuring that valuable information is not lost in the shared model.

FedMCSA [30]: First, the local model is decomposed at the client side, and model components are obtained at the server. Then, these model components undergo parallel self-attention operations to generate a complete personalized model, which is sent back to the clients. In this way, FedMCSA achieves full personalization in FL and promotes purposeful and efficient collaboration among clients.

pFedKT [31]: This method transfers historical local personalized knowledge from old local models to new current local models through a local supernetwork. It also transfers the latest global common knowledge from the latest global model to the new current local model through contrastive learning.

**Figure 10:**
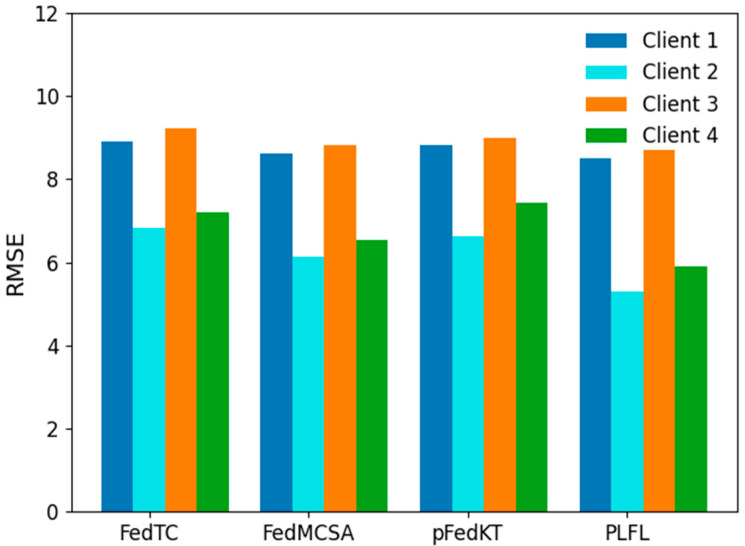
Comparison of prediction performance of different personalized federated learning methods on 4 clients.

Figure 10 presents the specific predictive performance metrics (RMSE) of several methods on four client devices after 50 communication rounds.

As observed from Figure 10, the PLFL (personalized lightweight federated learning model based on attention mechanism) exhibits a relatively balanced performance across different client devices, and its predictive performance surpasses that of other mainstream personalized federated learning methods. This is primarily attributed to its dynamic attention mechanism and optimized design for addressing sample imbalance issues. By introducing attention weights into the parameter aggregation of the global model, PLFL is capable of dynamically allocating weights based on the feature importance of each client, thereby enhancing personalized performance under uneven data distributions. The attention mechanism enables the global model to better capture key features of each client during aggregation, avoiding performance imbalance caused by the dominant weight of clients with large sample sizes. Furthermore, PLFL achieves a good balance between global and personalized performance by introducing an attention mechanism between clients and the server. The global model, while focusing on global consistency, also adapts to the data distribution characteristics of each client, thus improving predictive accuracy. This balance optimization results in PLFL having the lowest RMSE across all four clients, performing well regardless of whether the client has a large or small amount of data.

In contrast, other personalized federated learning methods such as FedTC, FedMCSA, and pFedKT also outperform baseline models but exhibit some deficiencies in terms of performance stability and generalization ability. For instance, FedTC retains personalized information through a dual-classifier mechanism but fails to effectively address the issue of sample imbalance among clients; FedMCSA performs well on some clients but lacks overall stability compared with PLFL; pFedKT enhances model generalization through a knowledge transfer mechanism but still has a higher prediction error on Client 3, indicating that there is room for improvement in balancing the global and local models.

In summary, PLFL’s performance is more superior and stable across different clients due to its dynamic weight allocation through the attention mechanism and balance optimization strategy. While other methods have also improved performance through their respective strategies, they still have certain limitations. This demonstrates that in federated learning, introducing mechanisms with strong adaptability, such as attention mechanisms, can better address issues of data heterogeneity and imbalance.

### 5.6. Discussion on Traffic Flow Prediction, Land Use, and Reflecting Human Activities

(a) Traffic flow prediction with land use: Traffic flow prediction serves as a valuable tool for reflecting the traffic demand and population density across different regions, which in turn helps to identify the core activity areas and potential development zones within a city. For instance, predictive analytics can uncover the traffic characteristics of various functional areas such as commercial districts, residential neighborhoods, and industrial zones, offering insights for the reference of land use functional zoning. If an area consistently experiences higher traffic flow than others over an extended period, it may suggest a higher commercial value, making it suitable for commercial land use planning. Conversely, areas with lower traffic flow but convenient access may be more appropriate for residential land use. Furthermore, traffic flow prediction can also reveal the strength of inter-regional connections; frequent traffic between certain areas may indicate a potential for collaboration or complementary functions, which could be prioritized for the construction of traffic corridors or for joint regional economic development. In addition, traffic flow prediction plays a crucial role in evaluating the feasibility and potential impact of land use planning proposals. During the planning phase of new developments or projects, simulating changes in traffic flow allows for the prediction of the planning’s impact on surrounding traffic, assessment of the rationality of road network loads, and determination of the necessity for additional transportation infrastructure, such as roads and public transportation stops. Moreover, the outcomes of traffic flow predictions also support the development of green cities. For example, by optimizing land use layout, it is possible to reduce traffic congestion and commuting distances, which can lead to decreased energy consumption and lower carbon emissions.
(b) Traffic flow prediction with human activities: Traffic flow prediction reflects the spatiotemporal distribution of vehicles and crowds, indirectly revealing the dynamic characteristics of human activities. Traffic data are significant indicators of human activity; for instance, peak commuting hours reflect the concentration of work-related travel, while changes in traffic around business districts or tourist attractions can manifest the intensity of shopping or tourism activities. Therefore, by forecasting traffic variations across different times and areas, it is possible to create heat maps of human activity, uncovering the functional attributes and dynamic changes of specific regions. This capability not only provides data support for studying human activity patterns but also helps identify hotspots of activity and potential traffic pressure points. Furthermore, traffic flow prediction offers vital guidance for traffic planning and urban management. In the short term, it can optimize real-time traffic control measures, such as adjusting traffic signal timing or dynamically allocating public transportation resources, to alleviate congestion during peak periods. In the long term, traffic flow prediction can provide insights into the growth trends of regional traffic, offering a reference for infrastructure expansion, road network optimization, and public transportation system planning. In conjunction with intelligent transportation systems, traffic flow prediction also supports autonomous driving route planning and the construction of smart cities, thereby enhancing the efficiency of urban operations and the quality of life for residents. Therefore, the integration of multi-source data and real-time analysis techniques will make traffic flow prediction even more significant in the application of human activity research and urban planning.

## 6. Conclusions

This paper proposes a personalized lightweight federated learning framework for short-term traffic flow prediction. Unlike the centralized training mode of machine learning, federated learning is a distributed training mode that offers the advantage of preserving data privacy while achieving performance comparable to centralized training models. Specifically, we first build a federated learning collaborative training framework. Then, to improve the communication efficiency of federated learning, we propose a dynamic model pruning strategy, which reduces the number of model parameters that need to be uploaded by pruning the local model of the client. Additionally, we address the challenge where clients with large amounts of data significantly impact the final global model, leading to a loss of personalization for other clients. To counteract this, we propose a federated learning personalization method that incorporates a feature attention mechanism in the global model parameter aggregation stage. By calculating the distance between the current local model parameters and the pre-processed global model parameters during aggregation, we can determine the differences between the models and customize different weights for each client model to improve personalization.

However, our study also faces several challenges. One major limitation is that the LPR data used in this research only represent traffic flow from a few road sections, which restricts the ability to effectively mine spatial features in traffic flow, thereby limiting the final prediction accuracy. Another issue is the lack of extensive discussion on the impact of the initial model on the overall prediction framework, which clearly influences the final prediction results. Additionally, this study does not consider the impact of external factors on traffic flow, such as adverse weather conditions and traffic accidents.

To address these challenges, future research directions may include the following:

1. Conducting network-level federated learning predictions, fully considering the spatial connectivity between each road section and intersection, to further improve prediction accuracy.
2. Utilizing different client initial models, as different machine learning models perform differently in traffic flow prediction. By selecting better initial models, the communication efficiency and accuracy of federated learning can be improved, thereby reducing the computational burden.
3. Fully considering the influence of external factors when inputting the model and exploring the extent to which each factor affects the final prediction accuracy.

In summary, our proposed framework demonstrates promising results in personalizing federated learning for traffic flow prediction but requires further refinement and consideration of additional factors to enhance its robustness and accuracy.

## Figures and Tables

**Table 1:** Explanation of formula symbols.

Formula Symbols	Explanation
C	Number of clients
$W_{n}^{s}$	Model parameters of the server during the nth communication
$W_{n+1}^{C}$	Model parameters for client c in n+1 communication
D(.,.)	Calculating the distance between two sets of neural parameters using the Euclidean distance formula
$A_{c}$	Importance weight for client model
$W^{l}$	Model parameters of server layer *l*
$W_{c}^{l}$	Model parameters for the c-th local client layer *l*

**Table 2:** Time spent on federated communication before and after model pruning.

	Total/s	Average Per-Round/s
Normal	565	11.3
SMP	360	7.2
DMP	385	7.7

**Table 3:** Comparison of prediction performance of different models (results represent 5 min, 15 min, and 30 min prediction performance, respectively).

	RMSE	MAE
LSTM	9.12/10.09/11.63	6.23/6.97/7.95
TGCN	8.43/8.93/9.51	5.59/5.95/6.58
DCRNN	7.86/8.03/8.22	4.89/5.01/5.32
STGCN	7.57/7.88/8.09	4.55/4.82/5.16
PLFL	7.11/7.63/8.21	4.05/4.79/4.98

## Data Availability

Data are available upon request by contacting the corresponding author.

## Appendix A

We utilize model parameter values, FLOPs (floating-point operations), and model prediction accuracy to represent the results of the dynamic pruning method DMP proposed in this paper. FLOPs refer to the total number of floating-point arithmetic operations (addition, subtraction, multiplication, division, etc.) performed by the model during runtime. It serves as a standard for measuring computational cost but does not equate to actual runtime, which also depends on hardware devices (such as the architecture of GPUs, memory bandwidth, etc.). Table A1 presents the results of model pruning.

**Table A1:** Results of model pruning.

	Parameters	FLOPs	MAE
Normal	$2.19\times 10^{6}$	$5.23\times 10^{8}$	4.12
SMP	$1.21\times 10^{6}$	$3.37\times 10^{8}$	5.06
DMP	$1.36\times 10^{6}$	$3.59\times 10^{8}$	4.18

SMP (static model pruning) typically employs a global fixed strategy during pruning, with the pruning structure of the model already determined after training, unable to adjust during the inference process based on the actual features of the input data. This “one-size-fits-all” pruning approach may lead to suboptimal performance of the model in certain complex scenarios (such as sudden traffic congestion or abnormal weather conditions), with noticeable decreases in accuracy. As seen in Table A1, the MAE (mean absolute error) after SMP pruning increases from 4.12 to 5.06, with an error increase of over 20%, indicating that it sacrifices considerable prediction accuracy while compressing the model.

In contrast, dynamic model pruning (DMP) is particularly suitable for tasks with high dynamics, such as traffic flow prediction. DMP can adjust the computational paths and activation channels of the network in real-time based on different input features, allowing the model to remain flexible in complex scenarios. For example, during the night when traffic flow is low or during off-peak periods with regular patterns, DMP can automatically prune some unnecessary computations to reduce computational overhead. In complex peak periods or during exceptional events, DMP can retain more of the network structure to more accurately capture the trends in traffic flow. This dynamic adaptability enables DMP to maintain a high level of prediction performance after pruning, with its MAE increasing only from 4.12 to 4.18, nearly matching the accuracy of the original model, while the parameter count and FLOPs are reduced by approximately 38% and 31%, respectively, achieving a good balance between efficiency and performance.
